# 基于高通量测序分析晚期肺癌患者肺部微生物多样性

**DOI:** 10.3779/j.issn.1009-3419.2020.103.16

**Published:** 2020-12-20

**Authors:** 卓楠 冉, 洁星 刘, 芬 王, 彩岩 信, 湘 沈, 山 曾, 章永 宋, 彬 熊

**Affiliations:** 1 646000 泸州，西南医科大学附属医院呼吸与危重症医学科 The Department of Pulmonary and Critical Care Medicine, The Affiliated Hospital of Southwest Medical University, Luzhou 646000, China; 2 646000 泸州，西南医科大学基础医学院病原生物教研室 Department of Pathogenic Biology, School of Basic Medical Sciences, Southwest Medical University, Luzhou 646000, China; 3 646000 泸州，西南医科大学公共实验技术中心分子生物学技术平台 Molecular Biotechnology Platform, Public Center of Experimental Technology, Southwest Medical University, Luzhou 646000, China

**Keywords:** 肺肿瘤, 基因组学, 生物多样性, 高通量测序, Lung neoplasms, Genomics, Biodiversity, High-throughput sequencing

## Abstract

**背景与目的:**

肺部微生物组与肺部疾病的发生密切相关，微生物可从多方面促进肿瘤的发生与发展。既往研究证实肺癌患者肺部微生物组较健康人发生了变化，但关于不同类型肺癌患者中肺部微生物组的微生物组成尚未明确。本研究旨在探讨不同组织学类型肺癌间的微生物组相关性及差异性。

**方法:**

采用Illumina HiSeq高通量测序技术对晚期肺癌患者痰样本进行细菌V3-V4区的16S rDNA测序及生物信息学分析。

**结果:**

经物种分类分析发现肺癌患者痰样本中以链球菌属、奈瑟氏菌属、普雷沃氏菌属为主。腺癌（adenocarcinoma, AD）组以链球菌属及奈瑟氏菌属为主，小细胞肺癌（small cell lung cancer, SCLC）组以奈瑟氏菌属为主，鳞状细胞癌（squamous cell carcinoma, SCC）组以链球菌属为主，混合型小细胞肺癌（combined small cell lung cancer, C-SCLC）组以链球菌属为主。

**结论:**

不同组织学类型肺癌的肺部细菌微生物组结构及组成存在差异性，不同组织学类型肺癌间优势菌群有所不同，本研究丰富了肺癌肺部细菌微生物组学数据。

## Abstract (en)

**Background and objective:**

The pulmonary microbiome is closely related to the occurrence of pulmonary diseases. The morbidity and mortality of lung cancer are relatively high in the world. It has been confirmed that lung microecology changes in lung cancer patients compared with healthy individuals. Furthermore, the abundance of some bacterial species shows obvious changes, suggesting their potential use as a microbial marker for the detection of lung cancer. The composition of the pulmonary microbiome in patients with different histological types of lung cancer has not been determined. We aim to study the correlation and difference of microbiome between different histological types of lung cancer.

**Methods:**

Illumina HiSeq high-throughput sequencing technology was used to sequenced the 16S rDNA V3-V4 region of bacterial in sputum samples of patients with advanced lung cancer.

**Results:**

It was found that *Streptococcus*, *Neisseria* and *Prevotella* were the main bacteria of lung cancer patients. Advantage bacterium group differ between different histological types of lung cancer. Adenocarcinoma (AD) group was dominated by *Streptococcus* and *Neisseria*, followed by *Veillonella*. Small cell lung cancer (SCLC) group was dominated by *Neisseria*, followed by *Streptococcus*. Squamous carcinoma (SCC) group was dominated by *Streptococcus*, followed by *Veillonella*. Combined small cell lung cancer (C-SCLC) group was dominated by *Streptococcus*, followed by *Prevotella*.

**Conclusion:**

The pulmonary bacterial microbiome of lung cancer of different histological types is different. This experiment enrichs the pulmonary bacterial microbiome data of lung cancer and fills the gap of pulmonary microbiome of small cell lung cancer.

随着高通量测序技术的发展，研究^[1]^发现健康人肺部存在大量微生物定植，一些无法通过传统培养法培养出的菌种可通过高通量测序检出。目前有研究^[2]^表明肺部微生物菌群与口腔菌群微吸入有关。既往多项研究证实肺部微生物菌群与呼吸系统疾病如哮喘^[3]^、慢性阻塞性肺疾病^[4, 5]^、肺囊性纤维化^[6]^等疾病的发生密切相关。

肺癌是一种恶性肿瘤，在世界范围内发病率、死亡率均较高，非小细胞肺癌（non-small cell lung cancer, NSCLC）患者5年生存率仅为21%，小细胞肺癌（small cell lung cancer, SCLC）患者5年生存率则仅为7%^[7]^。肺癌的临床症状出现晚，早期缺乏特异性筛查手段是其死亡率高的原因之一^[8]^。流行病学证据^[9]^表明，反复使用抗生素与增加肺癌风险之间存在关联，但肺部微生物菌群对肺癌的影响尚不清楚。有一项研究^[10]^表明肺癌患者化疗期间院内感染率可达44.77%。已有多项研究^[11-13]^证实肺癌患者肺部微生态较健康人群发生变化。但对于不同组织学类型肺癌之间肺部微生态差异性研究仍较少，特别是缺乏关于SCLC患者肺部微生态研究。

为明确不同组织学类型肺癌患者肺部微生物组成差异性，探讨临床变量与实验数据之间的相关性，筛选可能存在的肺癌微生物标志物，本研究采用Illumina HiSeq测序平台对晚期肺癌患者痰样本进行细菌16S rDNA的V3-V4区测序及生物信息学分析。

## 1 材料和方法

### 1.1 试剂及仪器

FastDNA SPIN kit试剂盒；二硫苏糖醇；甲醇；荧光剂；台式离心机；振荡器；水浴锅；电泳仪；凝胶成像系统；Illumina HiSeq高通量测序由北京百迈客公司完成。

### 1.2 受试者招募

招募西南医科大学附属医院呼吸与危重症医学科2018年10月-2019年9月收治的肺癌患者。纳入标准：①西南医科大学附属医院呼吸与危重症医学科收治的晚期肺癌患者；②年龄40岁-75岁；③组织学确诊肺癌；④所有患者对本研究知情同意。排除标准：①长期使用免疫抑制剂史；②胸腔手术史；③自身免疫性疾病病史；④气管插管、有创/无创机械通气操作史；⑤口腔疾病病史。

### 1.3 样本收集

嘱患者漱口清洁口腔后取痰样本于无菌杯中，将收集好的样本置于干冰保温盒内运送至实验室，放置于-80 ℃保存。统一将采集好的样本从-80 ℃冰箱取出，放置于冰上解冻。样本预处理：采用甲醇及二硫苏糖醇对采集好的痰样本进行预处理：30%甲醇5 mL+甲醇-二硫苏糖醇（30%甲醇1 mL+二硫苏糖醇0.24 g）500 μL溶解15 min，20, 000 rpm离心10 min，弃掉上清液，剩余物质用于DNA提取。

### 1.4 宏基因组的提取、保存及测序

采用FastDNA SPIN kit试剂盒对预处理的痰样本进行DNA提取（提取步骤详见试剂盒说明书）。使用80 μL DES洗脱保存DNA，将提取DNA进行电泳，电泳产生明亮条带则DNA提取成功。将提取DNA -20 ℃冰箱保存。将保存于-20 ℃的DNA同时上机测序：根据保守区设计得到引物，在引物末端加上测序接头，进行PCR扩增并对其产物进行纯化、定量和均一化，形成测序文库，建好的文库先进行文库质检，质检合格的文库用Illumina HiSeq 2500进行测序。高通量测序得到的原始图像数据文件，经碱基识别分析转化为原始测序序列，结果以FASTQ文件格式存储。

### 1.5 统计学分析

使用FLASH v1.2.7软件对每个样品的序列进行拼接，得到的拼接序列即原始数据；使用Trimmomatic v0.33软件，对拼接得到的原始数据进行过滤，使用UCHIME v4.2软件，鉴定并去除嵌合体序列，得到最终有效数据；使用QIIME（version 1.8.0）软件对序列的相似度为97%的水平下进行聚类、获得操作分类单元（operational taxonomic units, OTU），基于OTU分析结果，进行物种丰度统计；对数据进行样品多样性及差异性分析（分类学注释、Alpha多样性分析、Beta多样性分析、样品差异分析），发掘临床变量与实验数据之间的相关性。

## 2 结果

### 2.1 样本特点及测序质量

一共收集到18例肺癌患者合格痰样本18个（*n*=18），其中按照组织学分类：腺癌（adenocarcinoma, AD）患者样本6个，SCLC样本2个，鳞状细胞癌（squamous cell carcinoma, SCC）患者样本7个，混合型小细胞肺癌（combined-SCLC, C-SCLC）样本3个。所有患者中14例有吸烟史，4例有化疗药物治疗史，具体患者信息见表 1。18个样本共获得1, 065, 790条原始序列，拼接、过滤后共产生900, 016条优质序列，平均长度为424 bp。所有样本测序有效序列占测得序列数百分比均大于70%，所有样本平均序列长度415 bp-427 bp，质量值≥30的碱基占总碱基数的百分比为91.81%-94.83%，具体样本测序质量见表 2。

**表 1:** 入组患者的临床病例特征 Clinical and pathologic characters of enrolled patients

Feature	*n*	Percentage (%)
Gender		
Male	18	100
Female	0	0
Age (yr)		
≥60	13	72
< 60	5	28
Smoking history		
Yes	14	78
No	4	22
Chemotherapy history		
Yes	5	28
No	13	72
Classification		
AD	6	33
SCLC	2	11
SCC	7	39
C-SCLC	3	17
AD: adenocarcinoma; SCLC: small cell lung cancer; SCC: squamous cell carcinoma; C-SCLC: combined-SCLC.		

**表 2:** 样品各等级序列统计表 Statistics for each level of tags

Sample	Kindom	Phylum	Class	Order	Family	Genus	Species
A-1	29, 986	29, 986	29, 986	29, 986	29, 986	29, 986	29, 936
A-2	22, 407	22, 407	22, 407	22, 407	22, 407	22, 407	22, 376
A-3	42, 593	42, 593	42, 593	42, 593	42, 593	42, 593	42, 193
A-4	43, 947	43, 947	43, 947	43, 947	43, 947	43, 947	43, 895
A-5	42, 488	42, 488	42, 488	42, 488	42, 488	42, 488	42, 440
A-6	43, 633	43, 633	43, 633	43, 633	43, 633	43, 633	43, 490
B-1	34, 146	34, 146	34, 146	34, 146	34, 146	34, 146	33, 909
B-2	31, 801	31, 801	31, 801	31, 801	31, 801	31, 801	31, 711
C-1	28, 189	28, 189	28, 189	28, 189	28, 189	28, 189	28, 148
C-2	35, 189	35, 189	35, 189	35, 189	35, 189	35, 189	35, 046
C-3	35, 801	35, 801	35, 801	35, 801	35, 801	35, 801	35, 706
C-4	37, 149	37, 149	37, 149	37, 149	37, 149	37, 149	36, 778
C-5	45, 325	45, 325	45, 325	45, 325	45, 325	45, 325	45, 324
C-6	37, 118	37, 118	37, 118	37, 118	37, 118	37, 118	36, 997
C-7	36, 743	36, 743	36, 743	36, 743	36, 743	36, 743	36, 372
D-1	2, 519	2, 519	2, 519	2, 519	2, 519	2, 519	2, 517
D-2	21, 009	21, 009	21, 009	21, 009	21, 009	21, 009	20, 922
D-3	185, 416	185, 416	185, 416	185, 416	185, 416	185, 416	184, 393
A: AD; B: SCLC; C: SCC; D: C-SCLC.							

### 2.2 物种丰度统计

使用QIIME（version 1.8.0）软件中的UCLUST对序列在97%的相似度水平下进行聚类、根据序列不同的相似度水平，对所有序列进行OTU划分所有样本共获得371个OTU，其中SCLC样本OTU平均值最高，AD组次之，而样本量最大的SCC组OTU值最小。SCLC样本OTU平均值最高*n*=210.500，AD组*n*=204.667，C-SCLC组*n*=182.667，SCC组OTU值最小*n*=160.143。

根据OTU统计出各样品中各等级的注释到物种的序列数，最终得到每组样本的门（图 1A）、属（图 1C）、种（图 1E）平均数。18个样本一共发现物种216种，不同组织学类型肺癌组物种平均数AD组物种平均值最高，SCLC组次之，而SCC组物种平均值最小。不同组织学类型肺癌其主要细菌种类不同，AD组以厚壁菌门及拟杆菌门为主；SCC组及C-SCLC组以厚壁菌门为主，SCLC组以拟杆菌门为主，变形菌门次之（图 1B）。可见SCLC肺部菌落组成与其他类型肺癌有较大区别。属水平方面：AD组以链球菌属及奈瑟氏菌属为主，其次为韦荣氏球菌属，SCLC组以奈瑟氏菌属为主，其次为链球菌属，SCC组以链球菌属为主，其次为奈瑟氏菌属，C-SCLC组以链球菌属为主，其次为普雷沃氏菌属（图 1D），总体上，肺癌患者痰样本中链球菌属仍占主要地位。在种水平上，肺炎链球菌是肺癌患者痰样本中的主要菌种，其次为韦荣氏菌及奈瑟氏菌（图 1F）。

**图 1:**
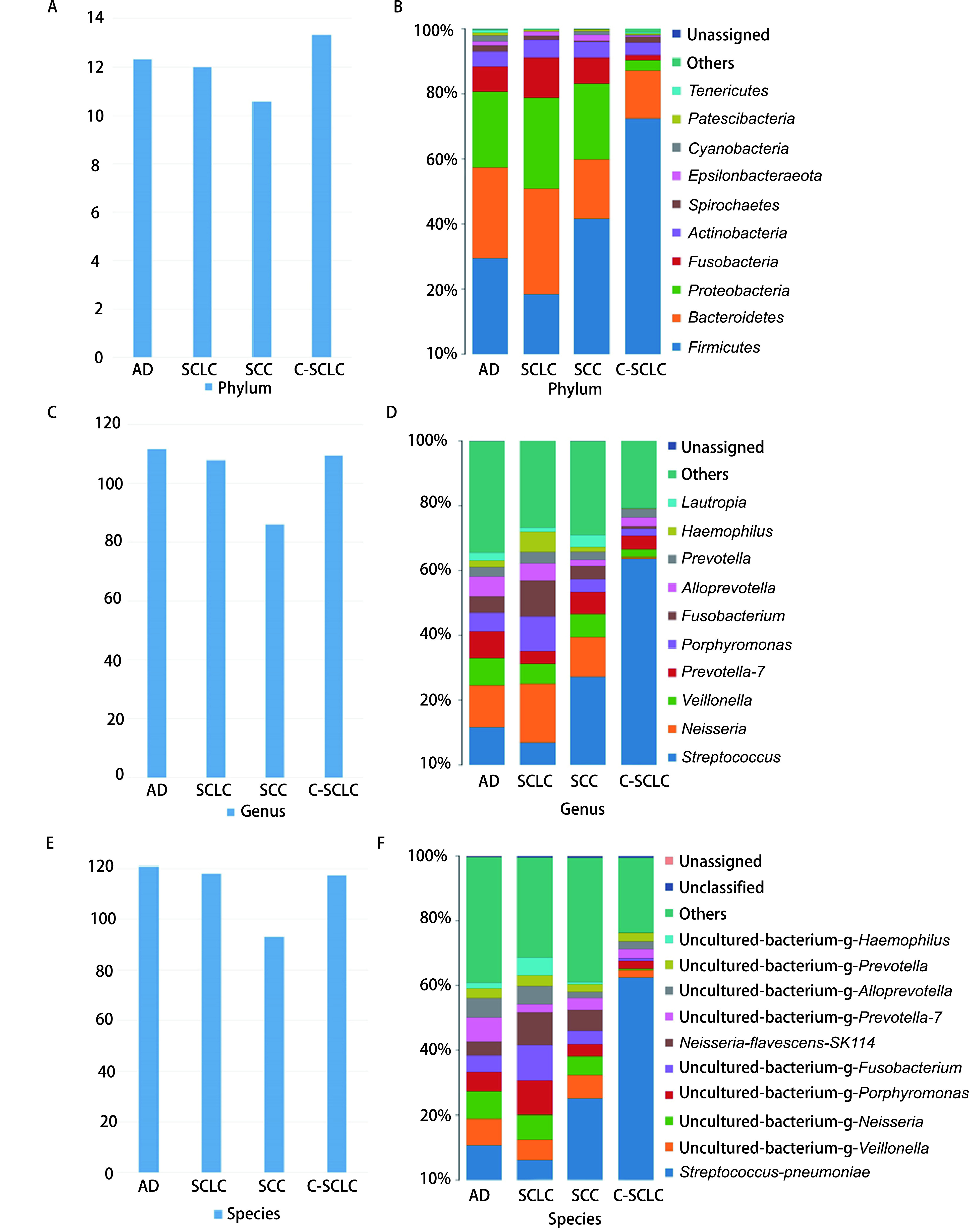
样品各等级物种柱状图。A：不同组别门水平（Phylum）物种平均数；B：不同组别门水平物种分布柱状图，一种颜色代表一个物种，色块长度表示物种所占相对丰度比例；C：不同组别属水平（Genus）物种平均数；D：不同组别属水平物种分布柱状图；E：不同物种种水平（Species）物种平均数；F：不同组别种水平物种分布柱状图。 Histogram of species distribution at each levels. A: The average of species at phylum level in different groups; B: Histogram of phylum horizontal distribution in different groups. Different color represents different species. The length of the color block represents the proportion of species in relative abundance; C: The average of species at genus level in different group; D: Histogram of genus horizontal distribution in different groups; E: The average of species at level species in different group; F: Histogram of species horizontal distribution in different groups.

**图 2:**
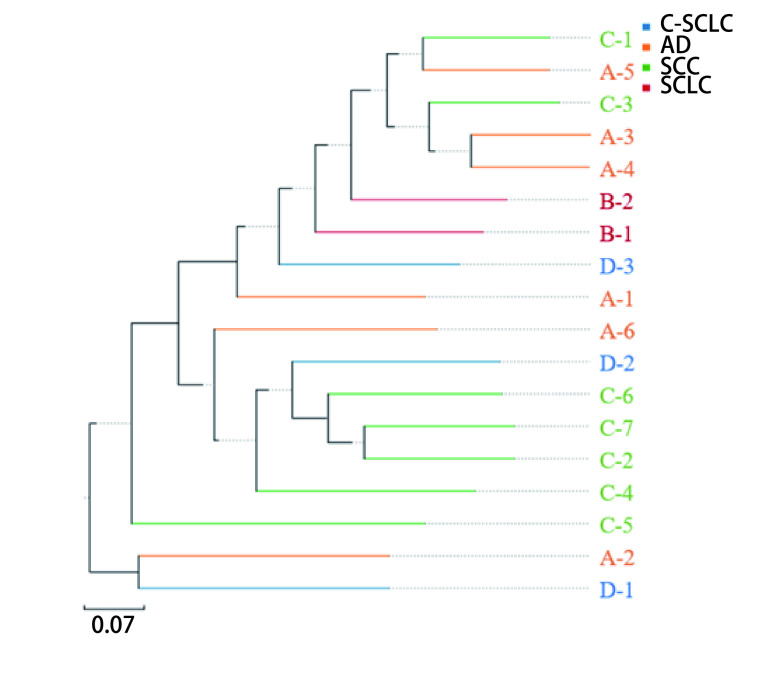
UPGMA分析图。样品越靠近，枝长越短，说明两个样品的物种组成越相似。不同颜色代表不同分组。 UPGMA analysis diagram. The closer of the samples means the species composition of the two samples is more similar. Different colors represent different groups.

### 2.3 多样性分析

使用Mothur（version v.1.30）软件，对样品Alpha多样性指数进行评估。结果显示：SCLC样本生物多样性最大，其次为AD，C-SCLC次之，而SCC样本生物多样性最小，详见表 3。

**表 3:** 各组样本Alpha多样性指数平均值 Average Alpha diversity index for each group

Item	AD	SCLC	SCC	C-SCLC
The average of Ace	230.48	239.42	184.48	204.09
The average Chao1	229.29	247.79	181.88	201.45
The Ace and Chao1 index are measures of species abundance. The higher the value, the greater the species abundance.				

使用QIIME软件对数据进行Beta多样性分析。采用非加权组平均法（unweighted pair-group method with arithmetic mean, UPGMA）对数据进行分析。结果显示：SCLC组内差异较小，C-SCLC组内差异较大，SCC与AD组间差异较大，C-SCLC与其他类型肺癌组组间差异最大，而SCLC标本与AD组间差异较小。

利用置换多元方差分析（Anosim）对不同分组的样品之间Beta多样性是否有显著差异进行检验。图 3显示*R*值为0.233，提示组间差异显著，C-SCLC与其他组织类型肺癌组差异尤其显著，AD最大值与最小值差值较小，且上四分位数与下四分位数差值较小，提示组内差异较小，而SCC组内差异较大（*P* < 0.05）。

**图 3:**
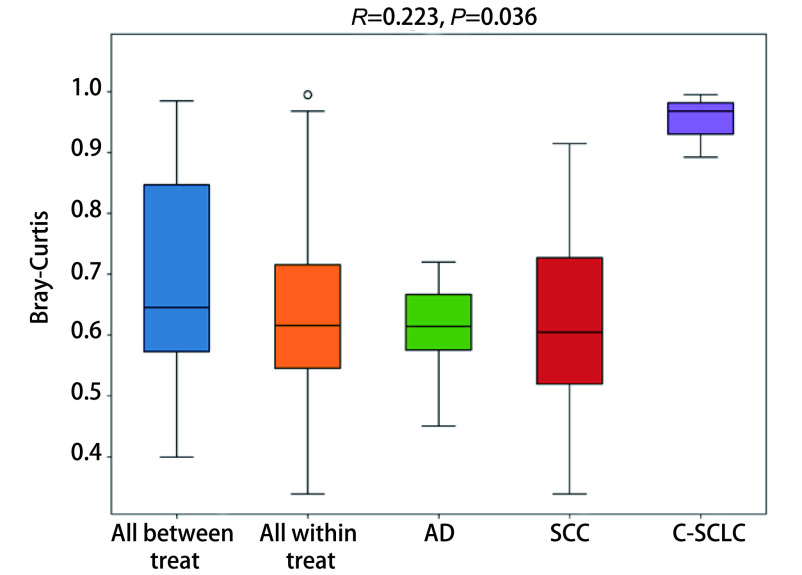
置换多元方差分析箱形图。Anosim分析得到的*R*值介于-1到1之间，越接近1表示组间差异越大于组内差异，*R* > 0提示组间差异显著，*P* < 0.05时说明检验的可信度高。纵坐表示Bray-Curtis距离；“All between treat”上方箱图代表所有组间样品Bray-Curtis距离数据，“All within treat”上方箱图代表所有组内样品Bray-Curtis距离数据，后面的箱型图分别是不同分组的组内样品间的Beta距离数据。SCLC因数据量少未纳入计算。 Anosim analysis box plot. The *R* value obtained by Anosim analysis was between -1 and 1. The closer to 1, the difference between groups was greater than that within groups. When *P* value is less than 0.05, the reliability of the test is high. Longitudinal seating represents the Bray-Curtis distance. The box diagram above "All Between Treat" represents the Bray-Curtis distance data of all between-treat samples. The box diagram above "All within Treat" represents the Bray-Curtis distance data of all within-treat samples. The box diagram behind represents the Beta distance data of samples in different groups. SCLC was not included in the calculation due to the small amount of data.

### 2.4 组间差异显著性分析

为研究两组样品间微生物群落丰度的差异，利用Metastats软件对组间的物种丰度数据进行*T*检验。结果显示：普雷沃氏菌属（*Prevotella*）、慢生根瘤菌（*Bradyrhizobium*）在AD与SCLC样本中丰度具有明显差异（*P* < 0.05），拟普雷沃菌属（*Alloprevotella*）、硫杆菌属（*Desulfovibrio*）在AD与SCC中丰度具有明显差异（*P* < 0.05），奈瑟菌属（*Neisseria*）、孪生球菌属（*Gemella*）、甲基杆菌属（*Methylobacterium*）在AD与C-SCLC中丰度具有明显差异（*P* < 0.05），普雷沃氏菌属（*Prevotella*）、鞘氨醇单胞菌属（*Sphingomonas*）、链球菌属（*Streptococcus*）在SCLC与SCC中丰度具有明显差异（*P* < 0.05），普雷沃氏菌（*Prevotella*）、沉积物杆状菌属（*Sediminibacterium*）在SCLC与C-SCLC中丰度具有明显差异（*P* < 0.05），沉积物杆状菌属（*Sediminibacterium*）、梭杆菌属（*Fusobacterium*）、奈瑟菌属（*Neisseria*）在SCC和C-SCLC中丰度具有明显差异（*P* < 0.05）。为寻找可能存在的不同肺癌类型的生物标志物，我们采用组间样品LEfSe分析[Line Discriminant Analysis (LDA) Effect Size]对不同分组数据进行分析筛选，C-SCLC中筛选出一种具有统计学意义的生物标志物*Candidatus terrybacteria*（图 4），该菌种具体在C-SCLC中发挥什么样的作用，其因果关系如何，它是否会加速病情的进展，这些问题仍待进一步研究证实。

**图 4:**
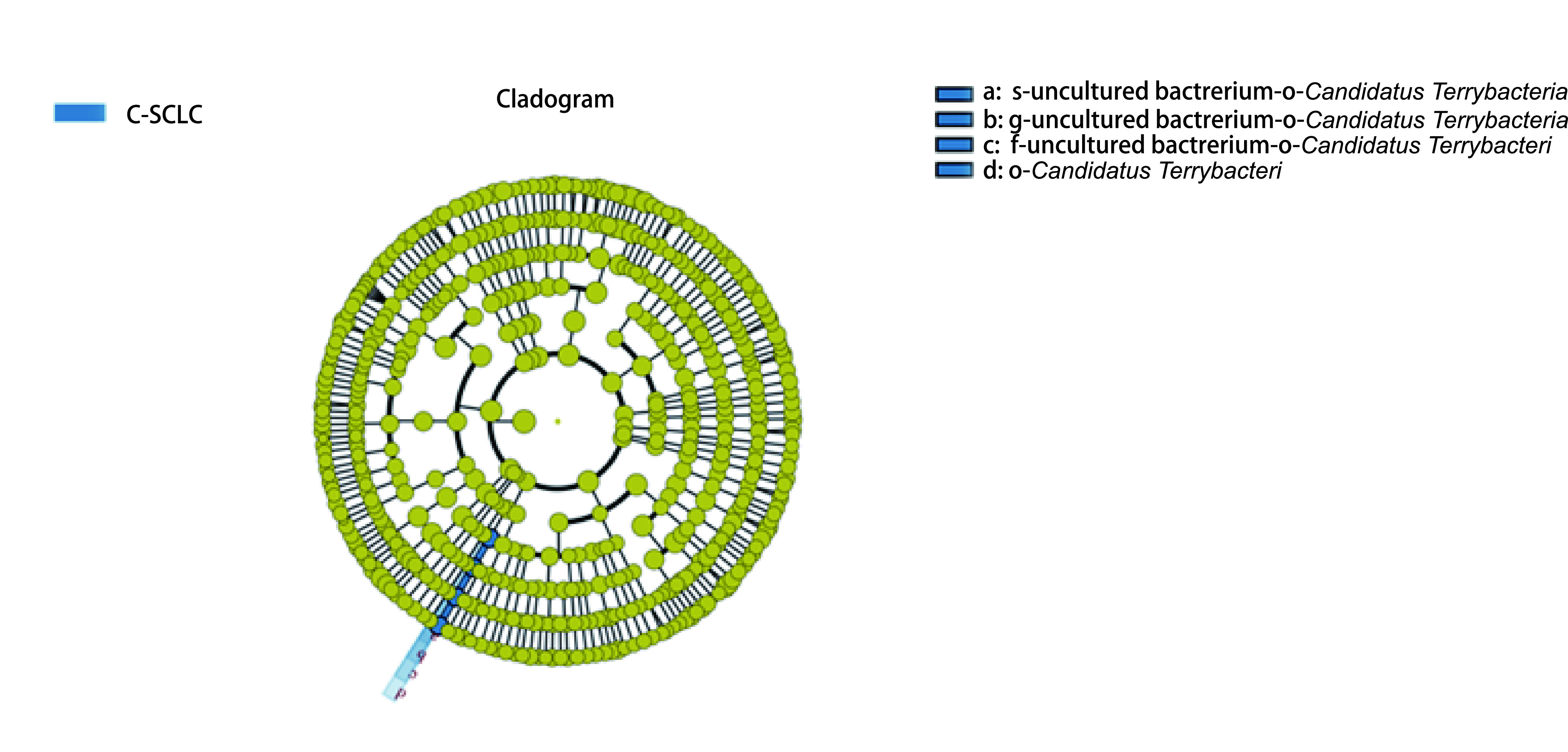
进化分支图。本图为C-SCLC测序数据通过LEfSe分析得出的进化分支图。由内至外辐射的圆圈代表了由门至种的分类级别；在不同分类级别上的每一个小圆圈代表该水平下的一个分类，小圆圈直径大小与相对丰度大小呈正比；着色原则为将无显著差异的物种统一着色为黄色，其他差异物种按该物种所在丰度最高的分组进行着色。 Cladogram. The Cladogram was obtained by LEfSe analysis of C-SCLC sequencing data. The circle from inside to outside represents the classification level from phylum to species. Each small circle represents a classification at the level. Species with no significant differences were uniformly colored yellow, while others were colored according to the group with the highest abundance of the species.

## 3 讨论

既往研究结果^[11-13]^表明：对肺癌患者非癌变部位和癌变部位、健康者肺部进行刷片检测，发现链球菌属在癌症病例中的含量明显高于健康组，奈瑟菌属在肺癌患者癌变部位也呈上升趋势。肺癌患者手术取得的肺癌组织样本中发现厚壁菌门（链球菌属）和拟杆菌门（普雷沃氏菌属）的丰度较正常样本明显升高。肺癌痰液样本中链球菌属丰度明显高于健康对照组。这与本实验结果有一致性，即肺癌患者肺部细菌微生物组中以链球菌属占优势，其次为奈瑟氏菌属。链球菌属在肺癌的发生发展中发挥怎样的作用？既往有肺部基础疾病病史如慢性阻塞性肺疾病、肺结核、肺炎的患者罹患肺癌的几率会增加^[14]^，有研究表明慢性呼吸系统感染可能会增加人体对致癌物的易感性，促进肺癌的发生^[15]^，而肺炎链球菌是常见的社区获得性肺炎的致病菌^[16]^，这提示链球菌在肺癌进程中可能发挥着一定的作用。

不同类型肺癌组微生物多样性不同，SCLC样本生物多样性最大，其次为AD，C-SCLC次之，而SCC样本生物多样性最小，C-SCLC与其他组织类型肺癌间物种多样性组间差异明显，而SCC物种多样性组内差异较大，这与疾病本身之间是否存在相关性及其因果关系现仍不明确，仍有待进一步研究证实。我们发现不同组织类型肺癌痰样本中的优势菌群不同，SCC样本链球菌属、硫杆菌属丰度高于AD，拟普雷沃氏菌属丰度低于AD，这与既往一项研究结果^[17]^相悖，其发现AD患者唾液样本中链球菌属和卟啉单胞菌属明显高于SCC患者，而SCC患者肺部微生物多样性明显低于AD。既往肺癌患者肺部微生物组学研究极少报道SCLC数据，而本研究纳入了SCLC样本，我们发现不同于其他组，SCLC以拟杆菌门丰度最大，变形菌门次之，SCLC中普雷沃氏菌属丰度明显大于其他组（*P* < 0.05）。C-SCLC是指肺癌患者肺组织病理检查为SCLC合并其他组织类型如AD、鳞癌、大细胞肺癌、大细胞神经内分泌癌^[18]^。既往没有关于C-SCLC的肺部微生态研究，本次通过Beta多样性分析发现C-SCLC组内差异较大，与其他组组间差异也较大，而C-SCLC是唯一一个组间样品LEfSe分析筛选出生物标志物的肺癌组。C-SCLC组织成分与SCLC有相似点，但其生物多样性及物种组成与SCLC均有较大差异。C-SCLC生物多样性明显低于SCLC，C-SCLC样本中沉积物杆状菌属（*Sediminibacterium*）及*Stomatobaculum*丰度高于SCLC，普雷沃氏菌属丰度低于SCLC（*P* < 0.05）。本实验仍存在不足，因SCLC及C-SCLC发病率较其他类型肺癌低，临床上患者数量较少，导致样本量不足，后续研究需加大样本量，减少抽样误差。

总之，本研究通过对18例晚期肺癌患者痰液进行高通量测序，发现不同组织学肺癌间微生物结构不同，且各自有其主要的微生物组成，其中SCLC及C-SCLC肺部微生物多样性为首次报道。本研究挖掘了不同组织学类型肺癌肺部微生物差异性，丰富了肺部微生物研究数据。
